# Solitary Colorectal Cancer Metastasis to the Pancreas

**DOI:** 10.1155/2019/4891512

**Published:** 2019-11-07

**Authors:** M. Karageorgou, D. Myoteri, T. Kotsis, G. Polymeneas, E. Bournakis, D. Dellaportas

**Affiliations:** ^1^2nd Department of Surgery, Aretaieion University Hospital, Medical School of Athens, Greece; ^2^Pathology Department, Aretaieion Hospital, Medical School, National and Kapodistrian University of Athens, Greece; ^3^Vascular Surgery Unit, Aretaieion Hospital, Medical School, National and Kapodistrian University of Athens, Greece; ^4^Oncology Unit, 2nd Department of Surgery, Aretaieio Hospital, Medical School, National and Kapodistrian University of Athens, V. Sophias 76, 11528 Athens, Greece

## Abstract

**Introduction:**

Secondary pancreatic metastasis from other solid organ malignancy is rare and accounts for less than 2% of all pancreatic tumors. The aim of this study is to highlight that colorectal metastatic disease in the pancreas could be in selected cases an indication for surgery rather than for palliative chemotherapy.

**Case Presentation:**

We present a case of a 62-year-old Caucasian female with a history of rectal adenocarcinoma. Four years ago, the patient underwent low anterior resection of the rectosigmoid, post neoadjuvant chemoradiotherapy, with adjuvant chemotherapy. During her follow-up, imaging examinations revealed a lesion in the pancreatic neck with features indicating primary pancreatic cancer. Near-total distal pancreatectomy with en bloc splenectomy was performed. Histopathology revealed metastatic disease compatible with colorectal adenocarcinoma as the primary cancer. Second-line chemotherapy was decided from the institutional tumor board. The patient remains disease free one year later.

**Conclusion:**

Pancreatic lesions in patients with a history of extrapancreatic malignancy should raise suspicions of metastatic disease. Surgical intervention is a legitimate treatment option for these pancreatic lesions, since they represent solitary disease deposits and of course in the context of multidisciplinary meeting decisions, and after proper and extensive staging investigations.

## 1. Introduction

A solid pancreatic lesion in tomographic imaging, in the majority of the cases, represents pancreatic adenocarcinoma. Demographically, pancreatic cancer is the fourth cause of death among cancer patients in the US, with high mortality rates [1]. Only 20% of the patients diagnosed with pancreatic adenocarcinoma are eligible for radical surgical treatment. Although rarely, malignant tumors originating from other primary sites can metastasize to the pancreatic gland. As primaries of the respiratory system, non-small cell lung cancer, gastrointestinal tract, kidney, breast, liver, ovary, urinary tract, and soft tissues can metastasize to the pancreas [2, 3]. It is very important to distinguish the exact nature of a pancreatic tumor as it entails a different treatment approach.

In order to distinguish the origin of a mass in the pancreas endoscopic ultrasound (EUS) guided FNA, cytopathology and immunohistochemistry assays play a crucial role [4]. The decision-making process about the treatment of a metastatic lesion in the pancreas is complex as it involves the identification of the primary cancer site, exclusion of other metastasis, taking into account the patient's performance status, and the surgical resectability of the lesion [5]. An interesting case of a colorectal (CRC) cancer patient with a solitary pancreatic metastasis, treated surgically, is presented herein.

## 2. Case Report

**Figure 1:**
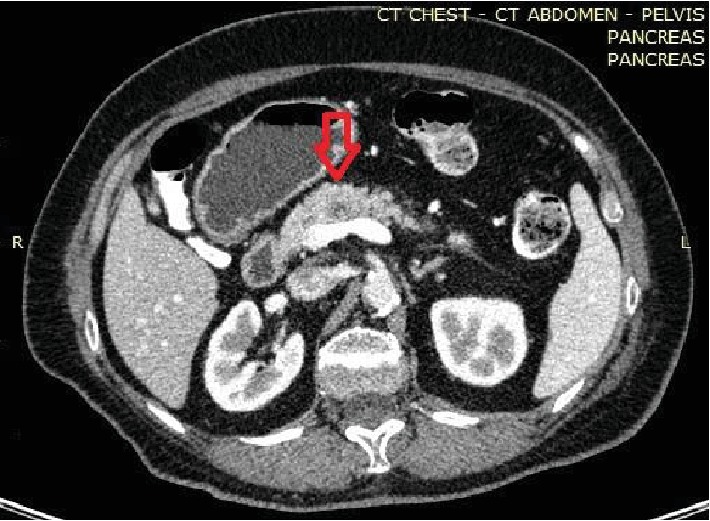
Computed tomography (CT image) demonstrating a hypodense pancreatic mass (red arrow) anterior to the superior mesenteric vein and splenic vein confluence.

**Figure 2:**
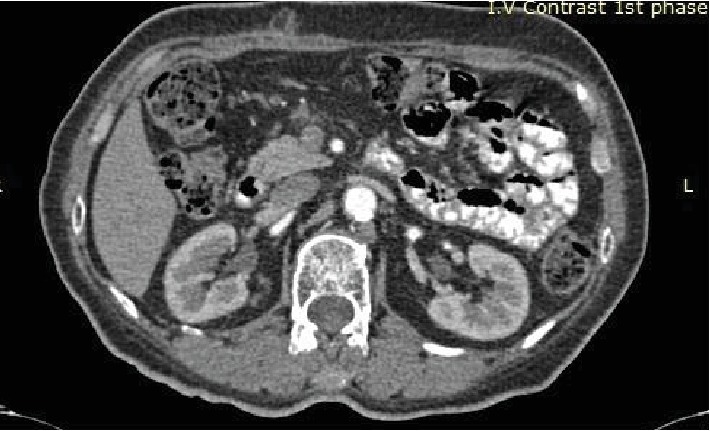
Follow-up computed tomography (CT image) with no evidence of recurrent disease.

A 62-year-old female with a history of rectal cancer four years ago presented with a pancreatic lesion identified during her annual follow-up. She has undergone a low-anterior resection of the rectosigmoid with total mesorectal excision (TME), post neoadjuvant chemoradiotherapy. Histopathology had shown a 3 cm rectal adenocarcinoma, grade II, staged as T4aN0M0 according to the AJCC/TNM 8^th^ Edition. She had also received six cycles of adjuvant chemotherapy with irinotecan, capecitabine, and bevacizumab. Two years later, the patient underwent wedge resections of lung metastasis in the right lower and left lower pulmonary lobes for metastatic CRC adenocarcinoma found in her follow-up, via video-assisted thoracoscopy (VATS). Four years after the first operation and almost two after the lung resections, the patient had an abdominal computed tomography (CT).

(see Figure 1) followed by a positron emission tomography (PET/CT), which revealed a 1.3 cm lesion in the neck of the pancreas with high ^18^FDG uptake (SUV-max = 5.8). Tomographic imaging could not differentiate between primary and secondary malignancies, and tumor markers were within a normal range. The lesion had no relation with the portal vein or the celiac/mesenteric vessels and was deemed technically operable. Multidisciplinary meeting decision opted for surgery considering the patient's long disease-free survival and good performance status in the absence of any other metastatic diseases. A near-total distal pancreatectomy with en bloc splenectomy was performed. On the 4th postoperative day, the patient developed a grade A pancreatic fistula, which was treated conservatively with total parenteral nutrition and somatostatin analogue. The patient was discharged on the 9^th^ postoperative day. Final histopathology report showed a 1.6 cm metastatic adenocarcinoma of CRC origin resected in clear surgical margins and 21 negative for metastatic disease peripancreatic lymph nodes. Immunohistochemistry examination was CK7(-), CK20(+), and CDX2(+). The hospital's tumor board decided for further adjuvant chemotherapy. The patient remains disease free one year later on her follow-up visits (see Figure 2).

## 3. Discussion

Colorectal cancer (CRC) is the second leading cause of morbidity due to malignancy in the US [6, 7]. It is well known that CRC is mainly surgically treated with adjuvant or neoadjuvant chemotherapy, radiotherapy, or intraoperative chemotherapy according to the individual patient's extent of disease and tumor stage.

Metastatic deposits to the pancreas are very rare and usually found in the context of disseminated disease. CRC is among the primaries that can metastasize to the pancreas which, however, accounts for 1.2% of all metastatic disease to this gland [8, 9]. The most common primary tumor sites metastasizing to the pancreas are renal cell carcinoma (RCC), breast cancer, melanoma, and gallbladder cancer whereas the most uncommon is seminoma [8, 9]. The patients usually are in their sixth decade of life and if symptomatic may develop obstructive jaundice or complain of diffuse abdominal pain [10]. Most lesions are found in tomographic imaging, usually with CT scan over the patient's follow-up or work-up if symptomatic. The echogeneity, location, and size of the metastatic lesions are not statistically significant in the differential diagnosis of a pancreatic lesion as stated by Geramizadeh et al. [11]. Biopsy of these pancreatic lesions is rarely needed and is performed only if it may change the oncologic management [12]. FNA combined with EUS is accounted for the highest specificity and accuracy results [13].

The proper surgical technique for metastasectomy in the pancreas depends on the localization of the mass and its size. Taking into account these factors, an enucleation or Whipple's procedure or a distal pancreatectomy with or without en bloc splenectomy can be performed. Atypical resections despite suggested previously seem to have higher complication rates than typical pancreatectomy procedures [3, 14]. Despite the anatomical and surgical challenges, there are very good results, with low mortality and morbidity rates in tertiary centers [10, 14]. Lymph node dissection could be omitted, as it is shown by Madkhali et al.; metastatic disease does not spread to the peripancreatic lymph nodes, which consequently reduces the extent of the surgical excision and the complications' rates [15]. Thus, metastatic pancreatic lesion resection is a safe procedure in tertiary centers [2, 5, 7, 8, 15]. According to the literature in centers of excellence, the rate of postoperative complications is the same as in surgeries performed for pancreatic primaries. As for RCC patients, significant efforts should be pursued through to detect metastatic disease in other sites, preoperatively and intraoperatively, suggesting in selected cases staging laparoscopy to avoid unnecessary laparotomy [14].

Nevertheless, surgical excision of metastatic disease in the pancreas has shown hopeful outcomes with longer disease-free and survival rates, especially in RCC primaries [10, 14]. It is also suggested that there is a better quality of life for patients that underwent surgical debulking. However, there is not enough evidence or randomized controlled trials in order to elucidate this matter. As it is stated according to Lee et al., RCC tumors are less aggressive and less invasive [6, 9, 14].

On the contrary, Madkhali et al. show that RCC primaries have higher recurrence rates at 3 years and the same overall survival and disease-free results [15].

## 4. Conclusions

This case report is aimed at pointing out that it should be always kept in mind that metastatic disease in the pancreas may appear in imaging tests similar to primary pancreatic cancer, particularly in the case of non-RCC primaries. The same stands for colorectal primaries and as a result jeopardizes the timely diagnosis and treatment of these patients. Furthermore, it should not be forgotten that surgical debulking and reducing the tumor load in CRC patients, even if it is metachronous, are proven to be effective and beneficial to these patients [5, 16–18].
